# Supplementary material for: Corticomuscular control of walking in older people and people with Parkinson’s disease
Source: Sci Rep. 2020 Feb 19;10:2980. doi: 10.1038/s41598-020-59810-w (PMC7031238; doi:10.1038/s41598-020-59810-w)
Supplement: Supplementary file 1 — Supplementary Information. [file 41598_2020_59810_MOESM1_ESM.pdf]

Supplementary Material

Corticomuscular control of walking in older people and people with Parkinson's disease

Authors: Luisa Roeder, Tjeerd W Boonstra, Graham K Kerr

Table S1 Main effect of condition and mean estimates of temporal gait parameters

Gait parameter	Overground		Treadmill		Condition effect	
	mean	SEM	mean	SEM	F	P
Walking speed (km/h)	4.05	0.065	4.04	0.065	1.03	0.31
Stride time (s)	**1.104**	**0.01**	**1.067**	**0.01**	**30.84**	**<0.001**
Stride time variability (s)	0.021	0.001	0.019	0.001	1.93	0.17
Step time (s)	**0.545**	**0.005**	**0.529**	**0.005**	**22.93**	**<0.001**
Step time variability (s)	0.011	0.001	0.011	0.001	0.002	0.96
Stance phase (s)	**0.697**	**0.008**	**0.676**	**0.008**	**15.84**	**<0.001**
Swing phase/ Single support (s)	**0.395**	**0.004**	**0.384**	**0.004**	**6.93**	**0.01**
Double support (s)	0.150	0.007	0.158	0.007	0.61	0.44

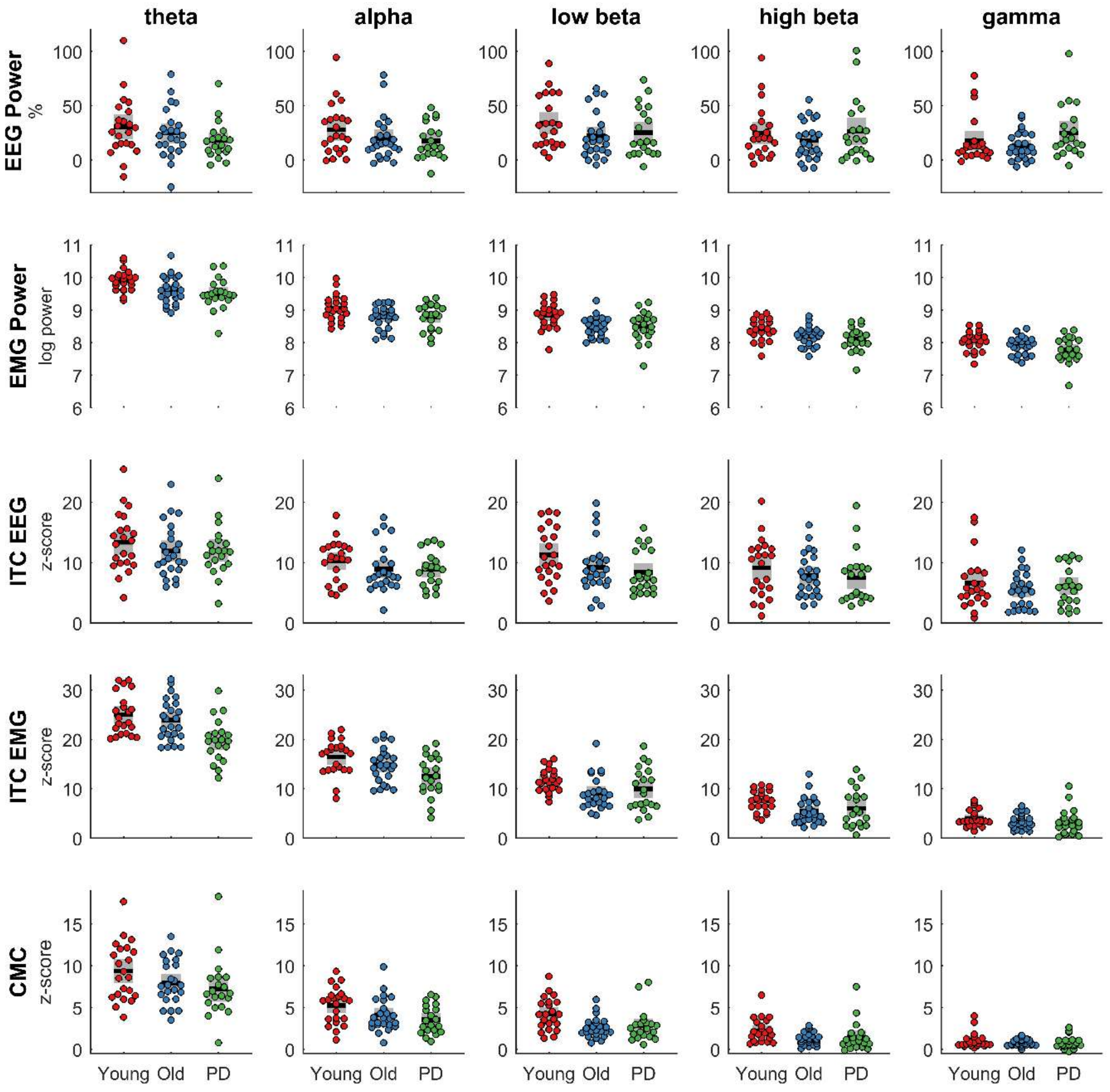

Figure S2. Data of group comparison for spectral measures during overground walking. Spectral measures (rows: EEG power, EMG power, ITC EEG, ITC EMG, CMC) were averaged over double support in different frequency bands (columns: theta, alpha, low beta, high beta, gamma) for each group (young, old, PD). Coloured dots show individual data of each participant, black horizontal lines show the group mean, grey boxes show the SEM. CMC, corticomuscular coherence; ITC, inter-trial coherence.

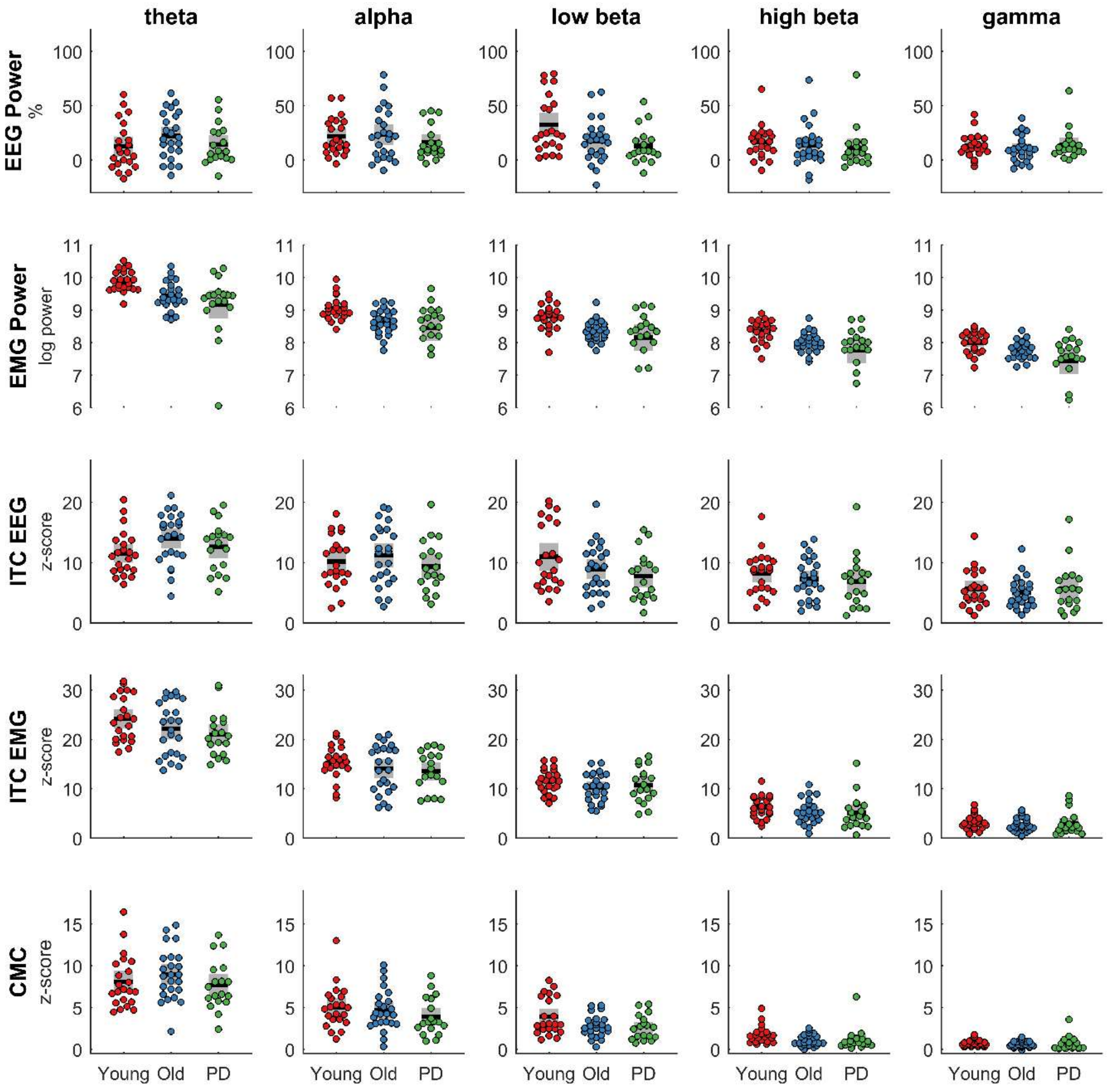

Figure S3. Data of group comparison for spectral measures during treadmill walking. Spectral measures (rows: EEG power, EMG power, ITC EEG, ITC EMG, CMC) were averaged over double support in different frequency bands (columns: theta, alpha, low beta, high beta, gamma) for each group (young, old, PD). Coloured dots show individual data of each participant, black horizontal lines show the group mean, grey boxes show the SEM. CMC, corticomuscular coherence; ITC, inter-trial coherence.

Table S4 Main effect of condition for spectral measures and mean estimates with 95% confidence intervals

Spectral measure	Overground		Treadmill		Condition effect		
	mean	95% CI	mean	95% CI	F	P	$P_{adjusted}$
**EEG power (%)**							
Theta	24.5	19.1-29.8	16.4	11.0-21.9	**5.53**	**0.020**	**0.042**
Alpha	21.8	16.8-26.8	21.1	16.0-26.2	0.07	0.79	0.82
Low beta	27.1	21.6-32.6	21.5	16.0-27.1	3.38	0.068	0.13
High beta	22.9	17.9-27.9	14.0	8.9-19.1	**11.1**	**0.001**	**0.004**
Gamma	18.7	14.8-22.5	12.8	8.9-16.7	**6.81**	**0.010**	**0.025**
**EMG power (log)**							
Theta	9.7	9.6-9.8	9.5	9.4-9.6	**11.5**	**0.001**	**0.004**
Alpha	8.9	8.8-9.0	8.7	8.6-8.9	**11.5**	**0.001**	**0.004**
Low beta	8.6	8.5-8.7	8.4	8.3-8.6	**13.8**	**<0.0005**	**0.004**
High beta	8.2	8.1-8.3	8.1	8.0-8.2	**19.8**	**<0.0005**	**0.004**
Gamma	7.9	7.8-8.0	7.7	7.6-7.8	**17.7**	**<0.0005**	**0.004**
**ITC EEG (z-score)**							
Theta	12.4	11.4-13.4	12.7	11.7-13.8	0.25	0.62	0.70
Alpha	9.4	8.4-10.3	10.3	9.4-11.3	2.99	0.085	0.15
Low beta	9.6	8.6-10.7	9.3	8.2-10.3	0.36	0.55	0.65
High beta	8.2	7.2-9.1	7.4	6.5-8.4	2.53	0.11	0.18
Gamma	6.0	5.3-6.8	5.4	4.6-6.2	2.30	0.13	0.20
**ITC EMG (z-score)**							
Theta	22.9	21.8-24.1	22.4	21.2-23.5	1.55	0.22	0.31
Alpha	14.6	13.7-15.6	14.3	13.4-15.3	0.43	0.51	0.64
Low beta	10.3	9.5-11.0	10.8	10.1-11.6	2.18	0.14	0.21
High beta	6.3	5.6-7.0	5.6	4.9-6.3	**7.02**	**0.009**	**0.025**
Gamma	3.5	3.1-4.0	2.9	2.4-3.4	**10.5**	**0.001**	**0.004**
**CMC (z-score)**							
Theta	8.2	7.4-8.9	8.2	7.5-9.0	0.02	0.89	0.89
Alpha	4.3	3.8-4.9	4.6	4.0-5.1	0.64	0.42	0.55
Low beta	3.2	2.8-3.6	3.1	2.7-3.5	0.18	0.67	0.73
High beta	1.7	1.4-1.9	1.3	1.0-1.6	**6.15**	**0.014**	**0.032**
Gamma	0.9	0.7-1.0	0.7	0.5-0.8	**6.92**	**0.009**	**0.025**

CI, confidence interval; ITC, inter-trial coherence; CMC, corticomuscular coherence.

Table S5 Post-hoc comparisons of significant group effects

Spectral measure	Healthy young		Healthy old		PD		P-value pairwise comparisons		
	mean	95% CI	mean	95% CI	mean	95% CI	Young-old	Young-PD	Old-PD
**EMG power (log)**									
Theta	9.9	9.7-10.1	9.5	9.3-9.7	9.3	9.1-9.5	**0.007**	**0.0002**	0.18
Alpha	9.1	8.9-9.2	8.7	8.5-8.9	8.6	8.4-8.8	**0.01**	**0.002**	0.5
Low beta	8.8	8.6-9.0	8.4	8.3-8.6	8.3	8.1-8.5	**0.004**	**0.001**	0.46
High beta	8.4	8.2-8.5	8.1	7.9-8.3	7.9	7.8-8.1	**0.02**	**0.001**	0.19
Gamma	8.0	7.9-8.2	7.8	7.7-8.0	7.6	7.4-7.8	0.07	**0.001**	0.08
**ITC EMG (z-score)**									
Theta	24.6	22.8-26.4	23.0	21.3-24.8	20.3	18.4-22.2	0.2	**0.002**	**0.04**
**CMC (z-score)**									
Low beta	4.1	3.5-4.7	2.7	2.1-3.2	2.7	2.1-3.3	**0.001**	**0.002**	0.9

CI, confidence interval; ITC, inter-trial coherence; CMC, corticomuscular coherence.

Table S6 Effect sizes (Cohen's d_s) of pairwise group comparisons

Spectral measure	Young-Old		Young-PD		Old-PD	
	d_s	95% CI	d_s	95% CI	d_s	95% CI
**EEG power (%)**						
Theta	0.11	-0.47, 0.69	-0.33	-0.95, 0.3	-0.44	-1.06, 0.18
Alpha	-0.18	-0.76, 0.40	-0.42	-1.05, 0.21	-0.24	-0.85, 0.38
Low beta	-0.70	-1.30, -0.10	-0.74	-1.37, -0.09	-0.02	-0.63, 0.59
High beta	-0.28	-0.90, 0.31	-0.10	-0.72, 0.53	0.18	-0.43, 0.79
Gamma	-0.29	-0.90, 0.30	0.34	-0.27, 0.97	0.64	0.00, 1.26
**EMG power (log)**						
Theta	-0.82	-1.42, -0.21,	-1.22	-1.89, -0.53	-0.41	-1.02, 0.21
Alpha	-0.79	-1.38, -0.18	-0.99	-1.60, -0.32	-0.20	-0.81, 0.41
Low beta	-0.89	-1.49, -0.28	-1.11	-1.78, -0.43	-0.22	-0.84, 0.39
High beta	-0.71	-1.31, -0.11	-1.12	-1.78, -0.44	-0.40	-1.02, 0.22
Gamma	-0.54	-1.13, 0.05	-1.09	-1.75, -0.41	-0.55	-1.17, 0.08
**ITC EEG (z-score)**						
Theta	0.16	-0.42, 0.74	-0.05	-0.68, 0.57	-0.22	-0.83, 0.40
Alpha	-0.05	-0.626, 0.53	-0.33	-0.96, 0.30	-0.29	-0.90, 0.33
Low beta	-0.60	-1.19, 0.00	-0.89	-1.54, -0.23	-0.28	-0.89, 0.33
High beta	-0.32	-0.90, 0.26	-0.45	-1.08, 0.19	-0.12	-0.73, 0.49
Gamma	-0.33	-0.91, 0.26	-0.10	-0.72, 0.53	0.24	-0.38, 0.85
**ITC EMG (z-score)**						
Theta	-0.37	-0.95, 0.22	-1.00	-1.66, -0.34	-0.64	-1.26, -0.01
Alpha	-0.42	-1.01, 0.17	-0.85	-1.50, -0.19	-0.42	-1.03, 0.20
Low beta	-0.71	-1.30, -0.11	-0.50	-1.13, 0.14	0.22	-0.39, 0.83
High beta	-0.58	-1.17, 0.01	-0.54	-1.17, 0.10	0.06	-0.55, 0.67
Gamma	-0.38	-0.96, 0.21	-0.31	-0.94, 0.32	0.07	-0.54, 0.68
**CMC (z-score)**						
Theta	-0.11	-0.69, 0.47	-0.53	-1.16, 0.11	-0.41	-1.03, 0.21
Alpha	-0.40	-0.98, 0.19	-0.78	-1.42, -0.13	-0.37	-0.99, 0.25
Low beta	-1.05	-1.67, -0.43	-1.01	-1.66, -0.34	-0.04	-0.65, 0.57
High beta	-0.78	-1.37, -0.17	-0.67	-1.30, -0.02	0.13	-0.49, 0.74
Gamma	-0.38	-0.96, 0.21	-0.28	-0.90, 0.35	0.11	-0.50, 0.72

CI, confidence interval; ITC, inter-trial coherence; CMC, corticomuscular coherence.

Table S7 Main effect of condition for EMG envelopes and mean estimates with 95% confidence intervals

**EMG envelope amplitude (%) at gait cycle phase**	**Overground**		**Treadmill**		**Condition effect**	
	mean	95% CI	mean	95% CI	F	P
Foot drop	57.1	52.7, 61.4	51.9	47.6, 56.3	**9.26**	**0.003**
Foot lift	58.1	55.1, 61.1	59.8	56.8, 62.9	2.42	0.12

CI, confidence interval.

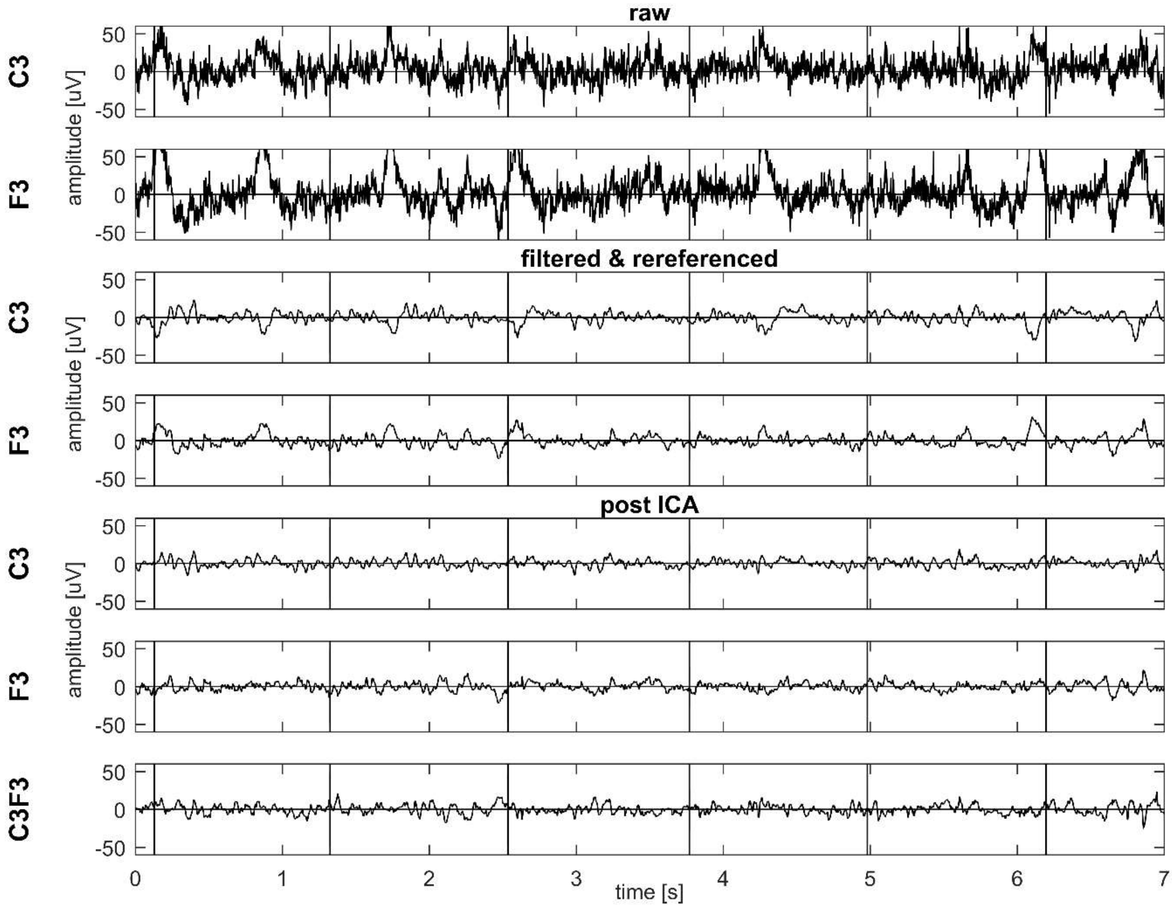

Figure S8. Data of one healthy older participant during overground walking. EEG signals of the C3 and F3 channels at different pre-processing steps: raw C3 and F3 signals (two top panels), after filtering and re-referencing (third and fourth panel), after Independent Component Analysis (ICA; fifth and sixth panel), bipolar signal C3-F3 (bottom panel). The vertical lines present heel strikes of the right foot.

EEG pre-processing and artefact removal

In addition to the pre-processing steps of the EEG signals presented in the main article (described in Data analysis), we also tested a number of alternative cleaning approaches, which are outlined below.

For each approach, we computed EEG power, EEG inter-trial coherence, and corticomuscular coherence of the bipolar EEG montages (C3-F3, C4-F4) and the contralateral tibialis anterior (TA) EMG signal of one healthy young participant during overground walking.

We tried six different cleaning approaches on the EEG signals in EEGLAB (v2019.0) and investigated their effects on the spectral estimates:

1. Band-pass filtering and re-referencing to a common average reference
2. Band-pass filtering, re-referencing and artefact subspace reconstruction (ASR) with a burst threshold of 20 (more conservative/lax)
3. Band-pass filtering, re-referencing and ASR with a burst threshold of 3 (more aggressive)
4. Band-pass filtering, re-referencing and ICA (extended Infomax algorithm), as described in the main article
5. Band-pass filtering, re-referencing, ASR (burst threshold at 20) and ICA
6. Band-pass filtering, re-referencing and adaptive mixture independent component analysis (AMICA)

Details about each approach and the resulting time-frequency spectra are described below.

1. Filtering and re-referencing

After EEG channels were visually inspected and segments with excessive noise (large-amplitude movement artefacts, EMG activity) were removed, EEG signals were then band-pass filtered (2nd order Butterworth, 0.5-70 Hz) and re-referenced to a common average reference. The resulting time-frequency graphs are shown below (S9).

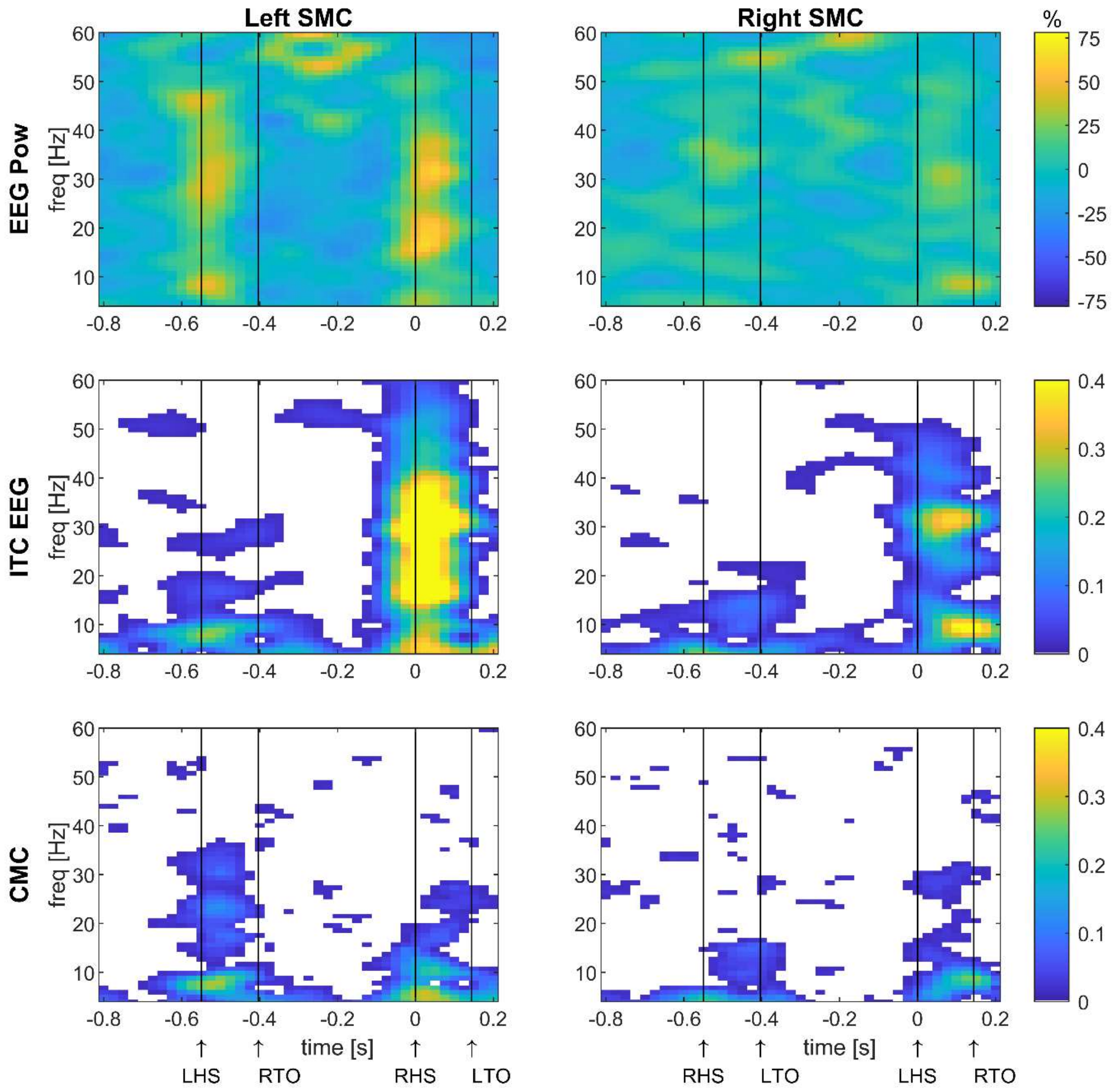

Figure S9. Time-frequency spectra after filtering and re-referencing of EEG signals. Event-related EEG power (EEG Pow, top row), inter-trial coherence of EEG (ITC EEG, middle row) and corticomuscular coherence (CMC, bottom row) acquired from bipolar EEG signals of the left (C3-F3) and right sensorimotor cortex (C4-F4) and EMG from the contralateral tibialis anterior (TA) of one healthy young participant during overground walking. EEG signals were first band-pass filtered and re-referenced to a common average before calculating the bipolar montages. EEG power shows the percent change from the average. Coherence values (ITC and CMC) are thresholded: average coherence values below the 95% CI are set to zero (white). The x-axis shows the time in seconds relative to heel strike (t=0) and the y-axis the frequencies in Hz. Black vertical lines indicate the footswitch events. LHS, left heel strike; LTO, left toe-off; RHS, right heel strike; RTO, right toe-off; SMC, sensorimotor cortex.

2. Filtering, re-referencing and artefact subspace reconstruction (ASR) with a burst removal threshold of 20

After band-pass filtering and re-referencing as outlined above in the first approach, we used artefact subspace reconstruction (ASR) to perform an automatic rejection of large movement artefacts. The threshold for burst removal was set to 20, as recommended in Chang et al. (2019) and applied in Wagner et al. (2019). This resulted in the following time-frequency spectra (S10).

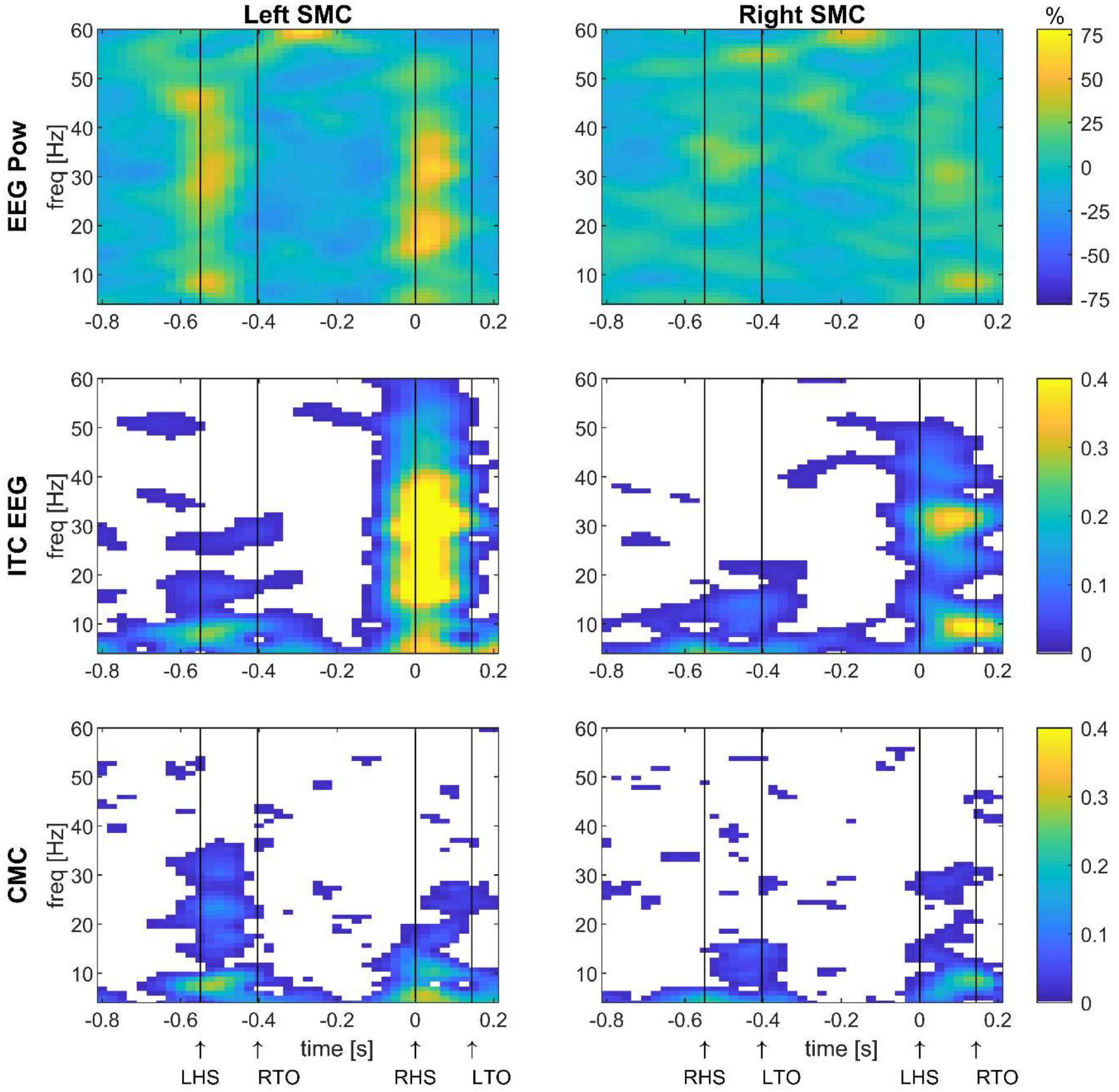

Figure S10. Time-frequency spectra after filtering, re-referencing and ASR (threshold 20) of EEG signals. Event-related EEG power (EEG Pow, top row), inter-trial coherence of EEG (ITC EEG, middle row) and corticomuscular coherence (CMC, bottom row) acquired from bipolar EEG signals of the left (C3-F3) and right sensorimotor cortex (C4-F4) and EMG from the contralateral tibialis anterior (TA) of one healthy young participant during overground walking. EEG signals were first band-pass filtered, re-

referenced to a common average, and processed by means of artefact subspace reconstruction (ASR) with a burst threshold of 20, before calculating the bipolar montages. EEG power shows the percent change from the average. Coherence values (ITC and CMC) are thresholded: average coherence values below the 95% CI are set to zero (white). The x-axis shows the time in seconds relative to heel strike (t=0) and the y-axis the frequencies in Hz. Black vertical lines indicate the footswitch events. LHS, left heel strike; LTO, left toe-off; RHS, right heel strike; RTO, right toe-off; SMC, sensorimotor cortex.

3. Filtering, re-referencing and ASR with a burst removal threshold of 3

We tried the same approach as in 2. (band-pass filtering, re-referencing, ASR), however for the ASR we set a more aggressive burst threshold at 3 (see figure S11). This threshold may be too aggressive and violate the balance between removing non-brain signals and retaining brain activities, as shown in Chang et al. (2019).

Notably, the resulting time-frequency spectra of the first three approaches (S9 to S11) are highly similar:

- post filtering + re-referencing,
- post filtering + re-referencing + lax ASR,
- post filtering + re-referencing + aggressive ASR.

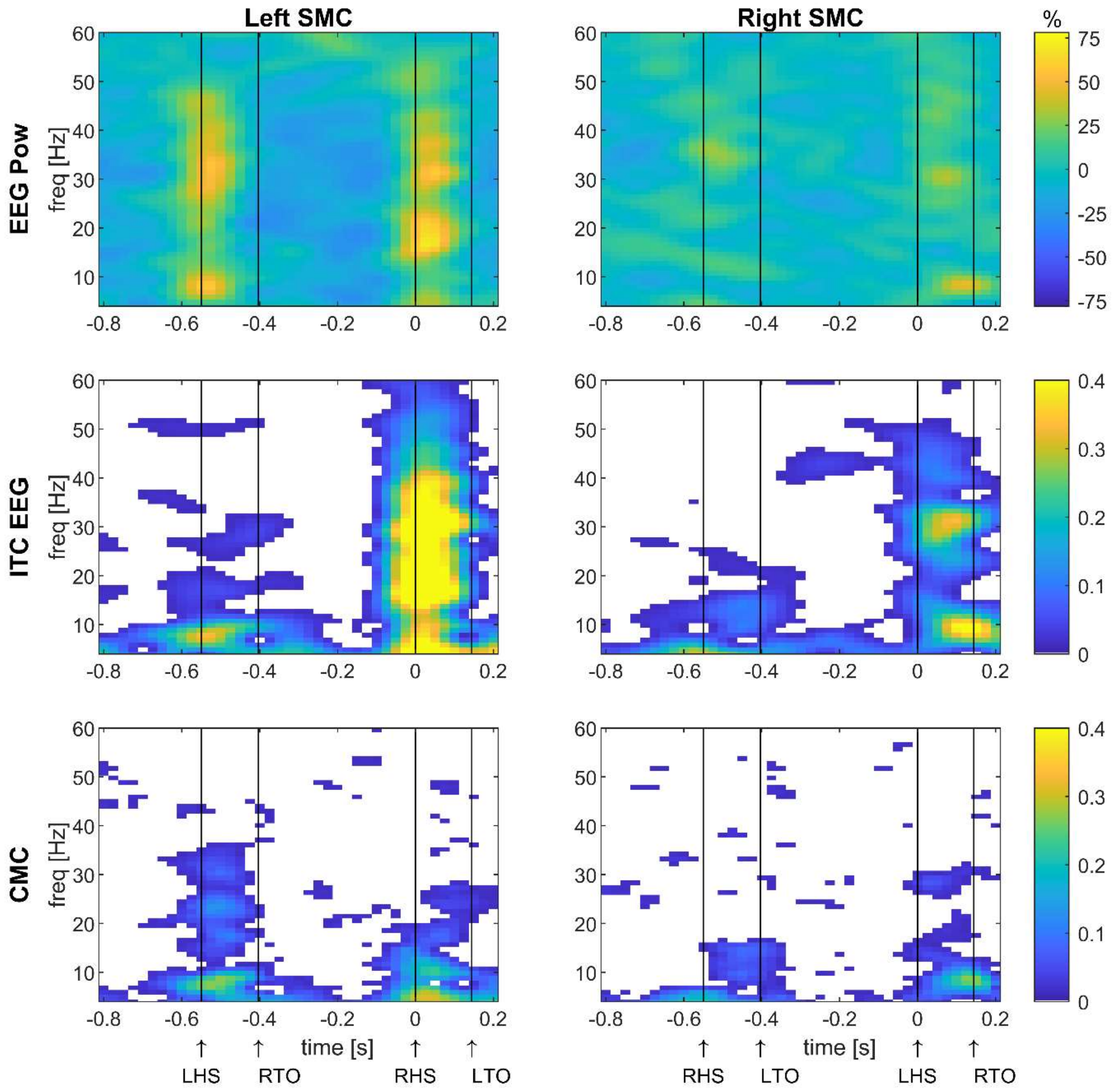

Figure S11. Time-frequency spectra after filtering, re-referencing and ASR (threshold 3) of EEG signals. Event-related EEG power (EEG Pow, top row), inter-trial coherence of EEG (ITC EEG, middle row) and corticomuscular coherence (CMC, bottom row) acquired from bipolar EEG signals of the left (C3-F3) and right sensorimotor cortex (C4-F4) and EMG from the contralateral tibialis anterior (TA) of one healthy young participant during overground walking. EEG signals were first band-pass filtered, re-referenced to a common average, and processed by means of artefact subspace reconstruction (ASR) with a burst threshold of 3, before calculating the bipolar montages. EEG power shows the percent change from the average. Coherence values (ITC and CMC) are thresholded: average coherence values below the 95% CI are set to zero (white). The x-axis shows the time in seconds relative to heel strike (t=0) and the y-axis the frequencies in Hz. Black vertical lines indicate the footswitch events. LHS, left heel strike; LTO, left toe-off; RHS, right heel strike; RTO, right toe-off; SMC, sensorimotor cortex.

4. Filtering, re-referencing and ICA (extended Infomax algorithm)

After band-pass filtering and re-referencing, we performed independent component analysis (ICA) using the extended infomax ICA algorithm implemented in EEGLAB (as outlined in the main article). Independent components containing eye blink, muscle, or movement artefacts were removed from the data, and subsequently time-frequency analysis was performed (S12).

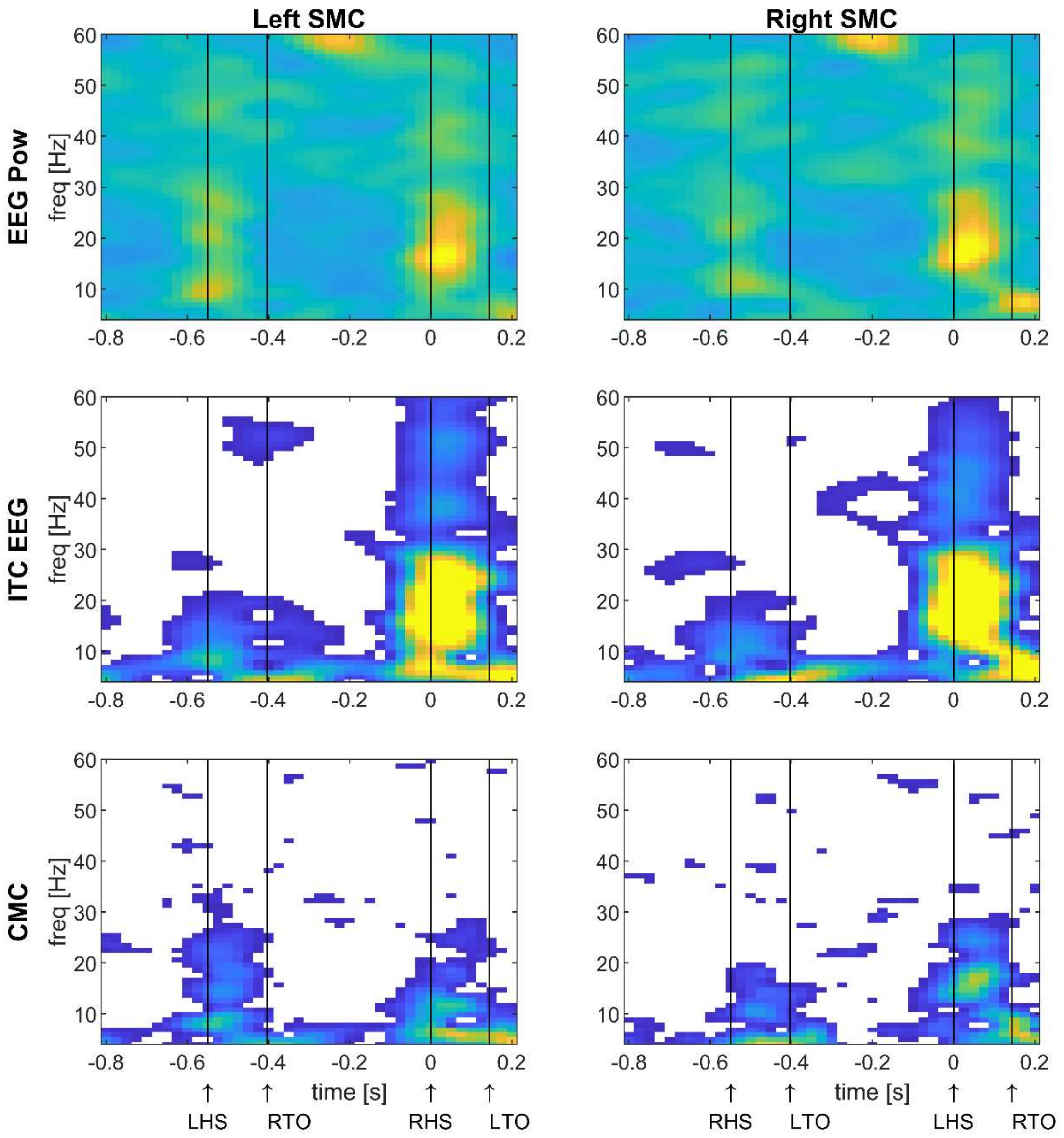

Figure S12. Time-frequency spectra after filtering, re-referencing and ICA of EEG signals. Event-related EEG power (EEG Pow, top row), inter-trial coherence of EEG (ITC EEG, middle row) and corticomuscular coherence (CMC, bottom row) acquired from bipolar EEG signals of the left (C3-F3) and right sensorimotor cortex (C4-F4) and EMG from the contralateral tibialis anterior (TA) of one healthy young participant during overground walking. EEG signals were first band-pass filtered, re-referenced to a common average, and processed by means of independent component analysis (ICA), before calculating the bipolar montages. EEG power shows the percent change from the average.

Coherence values (ITC and CMC) are thresholded: average coherence values below the 95% CI are set to zero (white). The x-axis shows the time in seconds relative to heel strike (t=0) and the y-axis the frequencies in Hz. Black vertical lines indicate the footswitch events. LHS, left heel strike; LTO, left toe-off; RHS, right heel strike; RTO, right toe-off; SMC, sensorimotor cortex.

5. Filtering, re-referencing, ASR and ICA

After band-pass filtering and re-referencing, we performed ASR with a burst removal threshold of 20. Subsequently, we performed ICA using the extended Infomax algorithm in EEGLAB. Chang et al. (2019) have shown that ICA decomposition is improved after ASR cleaning.

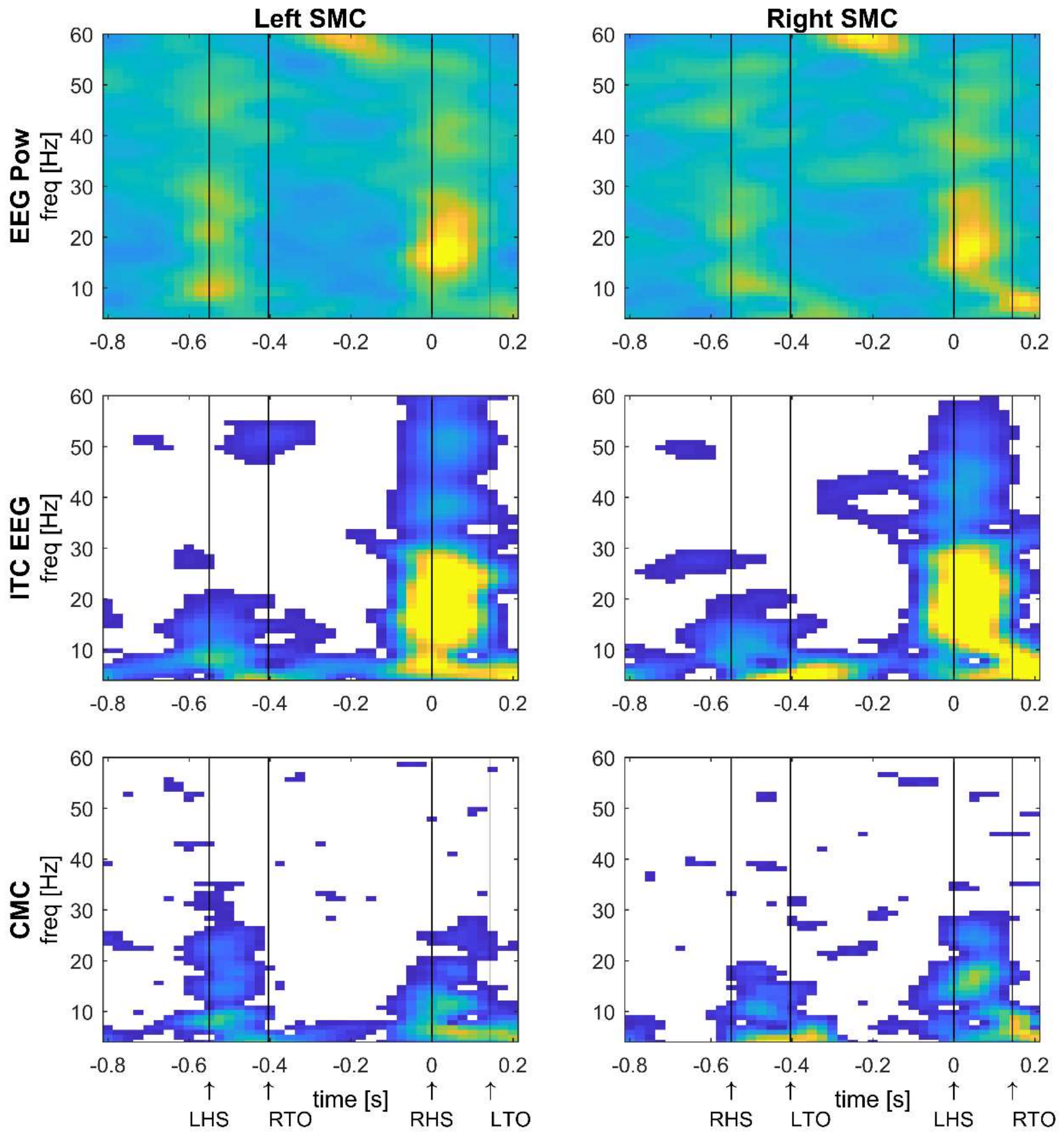

Figure S13. Time-frequency spectra after filtering, re-referencing, ASR and ICA of EEG signals. Event-related EEG power (EEG Pow, top row), inter-trial coherence of EEG (ITC EEG, middle row) and corticomuscular coherence (CMC, bottom row) acquired from bipolar EEG signals of the left (C3-F3) and right sensorimotor cortex (C4-F4) and EMG from the contralateral tibialis anterior (TA) of one healthy young participant during overground walking. EEG signals were first band-pass filtered, re-referenced to a common average, cleaned by ASR with a burst removal threshold of 20, and processed by means of independent component analysis (ICA), before calculating the bipolar montages. EEG power shows the percent change from the average. Coherence values (ITC and CMC) are thresholded: average coherence values below the 95% CI are set to zero (white). The x-axis shows the time in seconds relative to heel strike (t=0) and the y-axis the frequencies in Hz. Black vertical lines indicate the footswitch events. LHS, left heel strike; LTO, left toe-off; RHS, right heel strike; RTO, right toe-off; SMC, sensorimotor cortex.

6. **Filtering, re-referencing and adaptive mixture independent component analysis (AMICA)**

After band-pass filtering and re-referencing, we performed adaptive mixture ICA (AMICA). Subsequently, independent components containing eye blink, muscle, or movement artefacts were removed from the data, and time-frequency analysis was performed.

AMICA has been shown to be the best performing decomposition algorithm, although other decomposition methods (e.g. ICA based on the extended Infomax algorithm) have been shown to yield similar components (Delorme et al. 2012). The model order of AMICA has been shown not to critically affect the decomposition (Hsu et al. 2018).

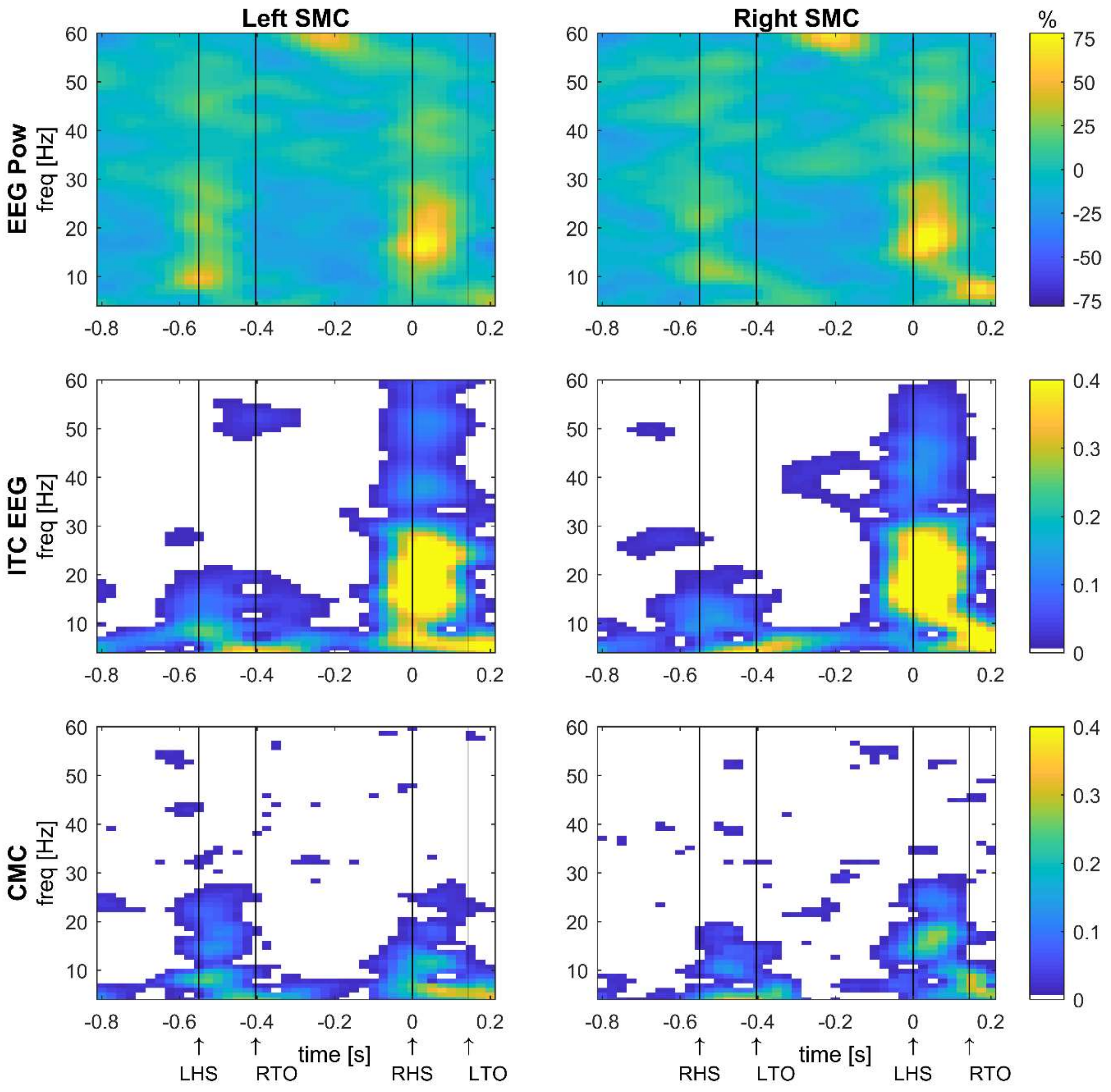

Figure S14. Time-frequency spectra after filtering, re-referencing and AMICA of EEG signals. Event-related EEG power (EEG Pow, top row), inter-trial coherence of EEG (ITC EEG, middle row) and

corticomuscular coherence (CMC, bottom row) acquired from bipolar EEG signals of the left (C3-F3) and right sensorimotor cortex (C4-F4) and EMG from the contralateral tibialis anterior (TA) of one healthy young participant during overground walking. EEG signals were first band-pass filtered, re-referenced to a common average, and processed by means of adaptive mixture independent component analysis (AMICA), before calculating the bipolar montages. EEG power shows the percent change from the average. Coherence values (ITC and CMC) are thresholded: average coherence values below the 95% CI are set to zero (white). The x-axis shows the time in seconds relative to heel strike (t=0) and the y-axis the frequencies in Hz. Black vertical lines indicate the footswitch events. LHS, left heel strike; LTO, left toe-off; RHS, right heel strike; RTO, right toe-off; SMC, sensorimotor cortex.

Notably, the resulting time-frequency spectra of the last three approaches (S12 to S14) trialled herein are highly similar:

- post filtering + re-referencing + ICA,
- post filtering + re-referencing + ASR + ICA,
- post filtering + re-referencing + AMICA.

In summary, the main finding of this study (peak in low beta-band power, inter-trial coherence and corticomuscular coherence) is not clearly visible in the time-frequency spectra after filtering and re-referencing ±ASR (especially over the right sensorimotor cortex in this participant). In contrast, the low beta peak becomes discernible after filtering, re-referencing +ICA, +ASR+ICA, and +AMICA. Importantly, extended Infomax ICA with or without preceding ASR, and AMICA led to almost identical time-frequency results. This suggests that artefacts present in the raw data may prevent the detection of this low beta peak, which is only evident after a form of ICA cleaning (for the shown dataset it appears that artefacts mainly affected the right sensorimotor cortex and less the left cortex, as the time-frequency results for the left sensorimotor cortex remain largely unchanged after ICA cleaning). Moreover, it suggests that ASR only (without subsequent ICA) is not sufficient to adequately clean the data, despite the low number of channels recorded in this study.

Time-frequency analyses of independent components

We performed time-frequency analyses on the 10 independent components (IC) that were identified by ICA (extended Infomax), as per pre-processing approach described in the main article. Below we present EEG power, EEG inter-trial coherence, and corticomuscular coherence of the 10 ICs and the left tibialis anterior (TA) EMG signal (S15 to S17) of one healthy young participant during overground walking (same dataset as above outlining the six different pre-processing approaches). Note that IC 1 to 3 were removed from this dataset, as they were identified as motion artefacts and eye movements, and IC 4 to 10 were projected back onto the channels before complete the time-frequency analyses reported in the main article.

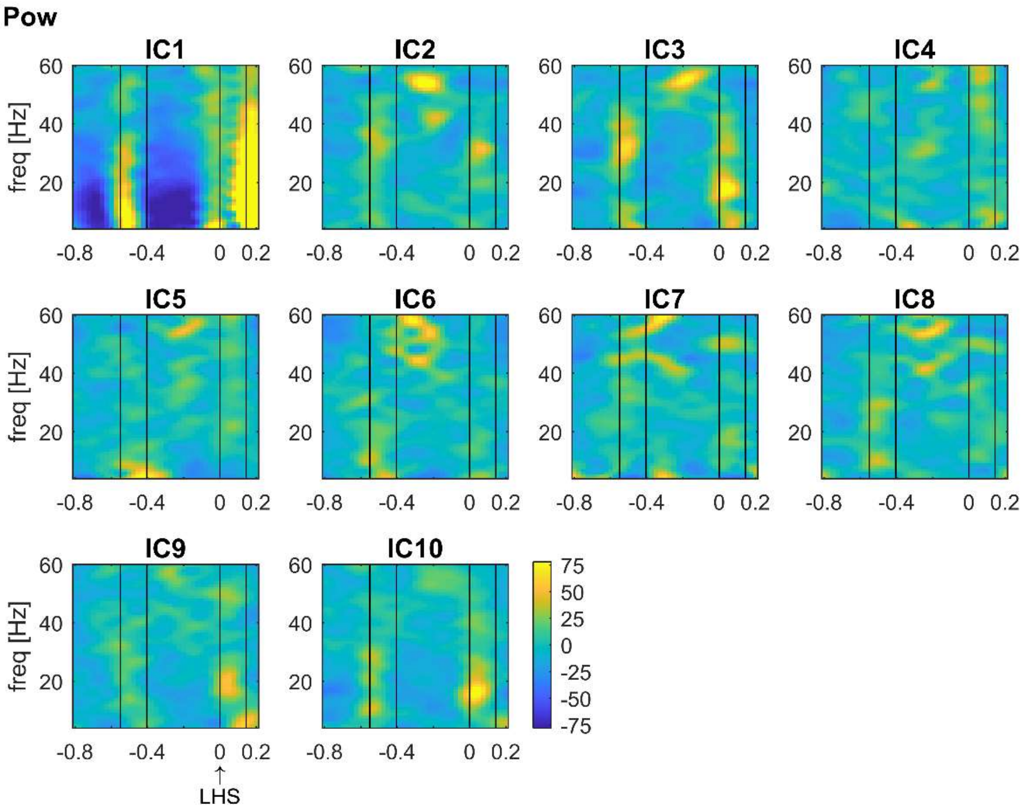

Figure S15. Event-related power of independent component signals. ICs after extended Infomax decomposition of EEG signals of one healthy young participant during overground walking. IC power shows the percent change from the average. The x-axis shows the time in seconds relative to heel strike of the left foot (t=0) and the y-axis the frequencies in Hz. Black vertical lines indicate the other footswitch events (toe-off and heel strike of both feet). IC, independent component; LHS, left heel strike.

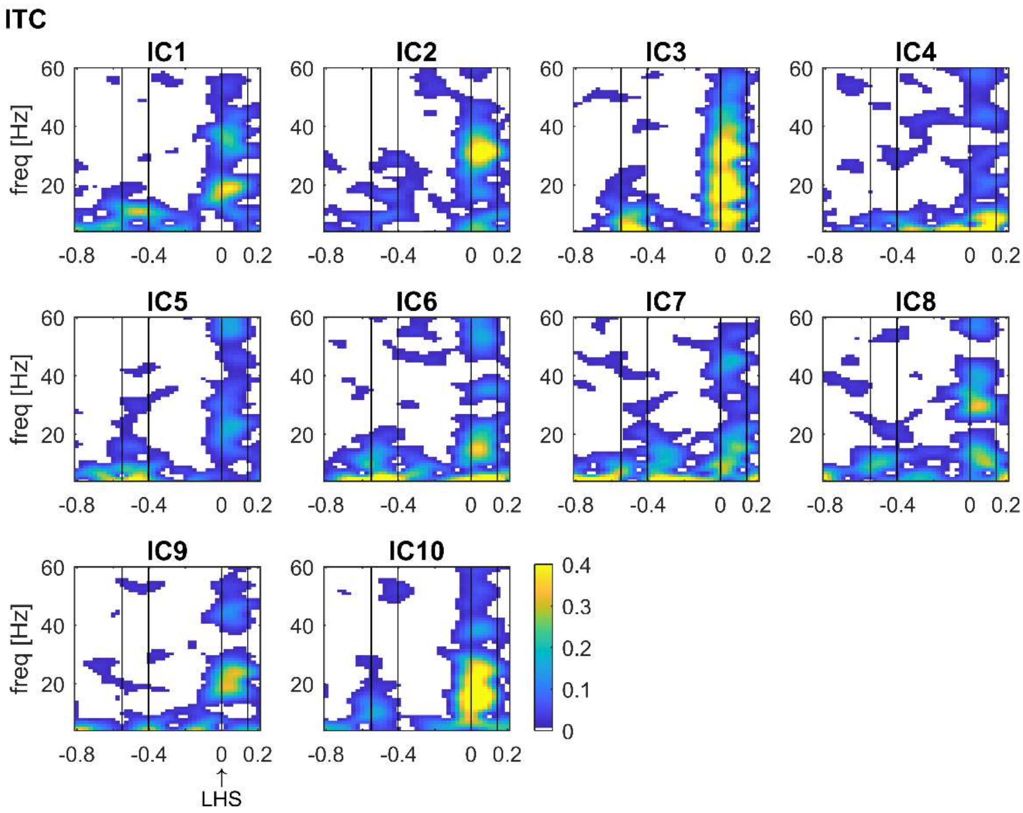

Figure S16. Inter-trial coherence of independent component signals. ICs after extended Infomax decomposition of EEG signals of one healthy young participant during overground walking. ITC values are thresholded: average coherence values below the 95% CI are set to zero (white). The x-axis shows the time in seconds relative to heel strike of the left foot (t=0) and the y-axis the frequencies in Hz. Black vertical lines indicate the other footswitch events (toe-off and heel strike of both feet). IC, independent component; LHS, left heel strike.

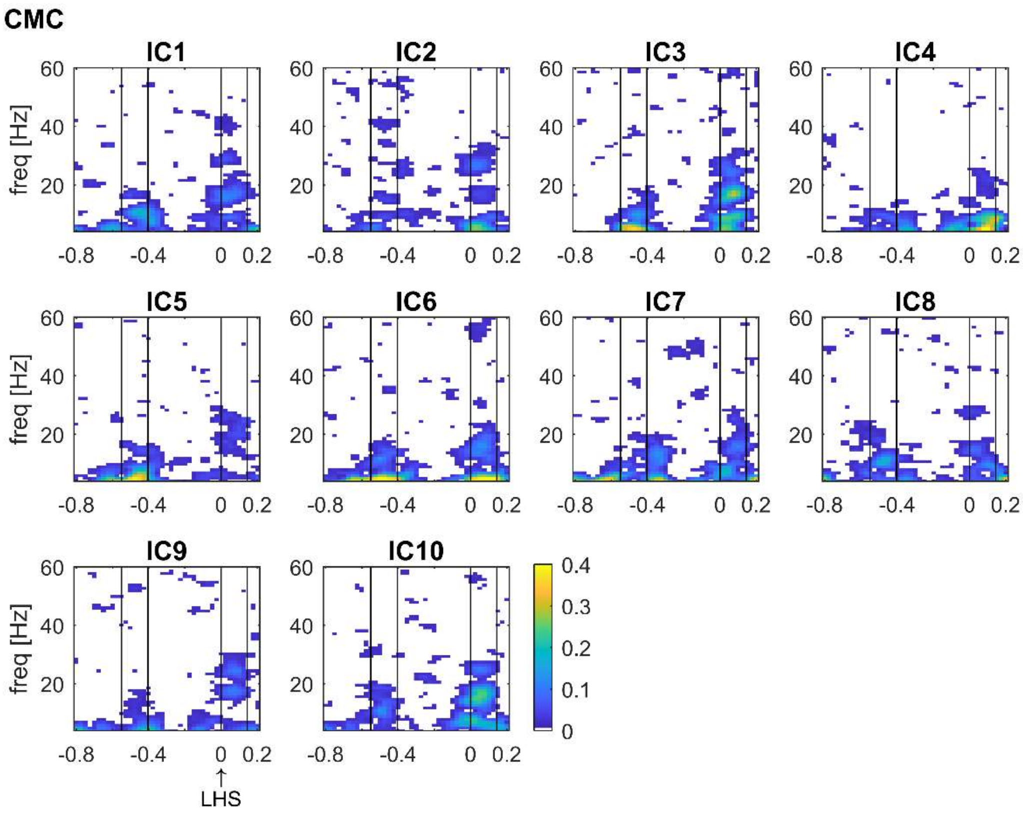

Figure S17. Corticomuscular coherence of independent component signals. ICs after extended Infomax decomposition of EEG signals and EMG from the left tibialis anterior (TA) of one healthy young participant during overground walking. CMC values are thresholded: average coherence values below the 95% CI are set to zero (white). The x-axis shows the time in seconds relative to heel strike of the left foot (t=0) and the y-axis the frequencies in Hz. Black vertical lines indicate the other footswitch events (toe-off and heel strike of both feet). IC, independent component; LHS, left heel strike.

These results suggest some mixing of sources (artefacts and brain activity). That is, both IC3 and IC10 show a peak of corticomuscular coherence in the beta band, while IC3 was identified as artefact component and IC10 was identified as brain component. These results may also indicate that our cleaning was conservative and that ICs containing activity from brain sources were identified as artefact components.

Time-frequency analyses of artefact components

We performed time-frequency analyses on the EEEG-artefact components that were identified by ICA (extended Infomax). To this end, we excluded all EEG components and kept the artefact components, which were projected back onto the channels. Subsequently, we performed time-frequency analysis on these signals. Below we present EEG power, EEG inter-trial coherence, and corticomuscular coherence of these artefact signals of the right sensorimotor cortex (C4-F4) and the left tibialis anterior (TA) EMG during overground walking in the young, old and PD groups (S18).

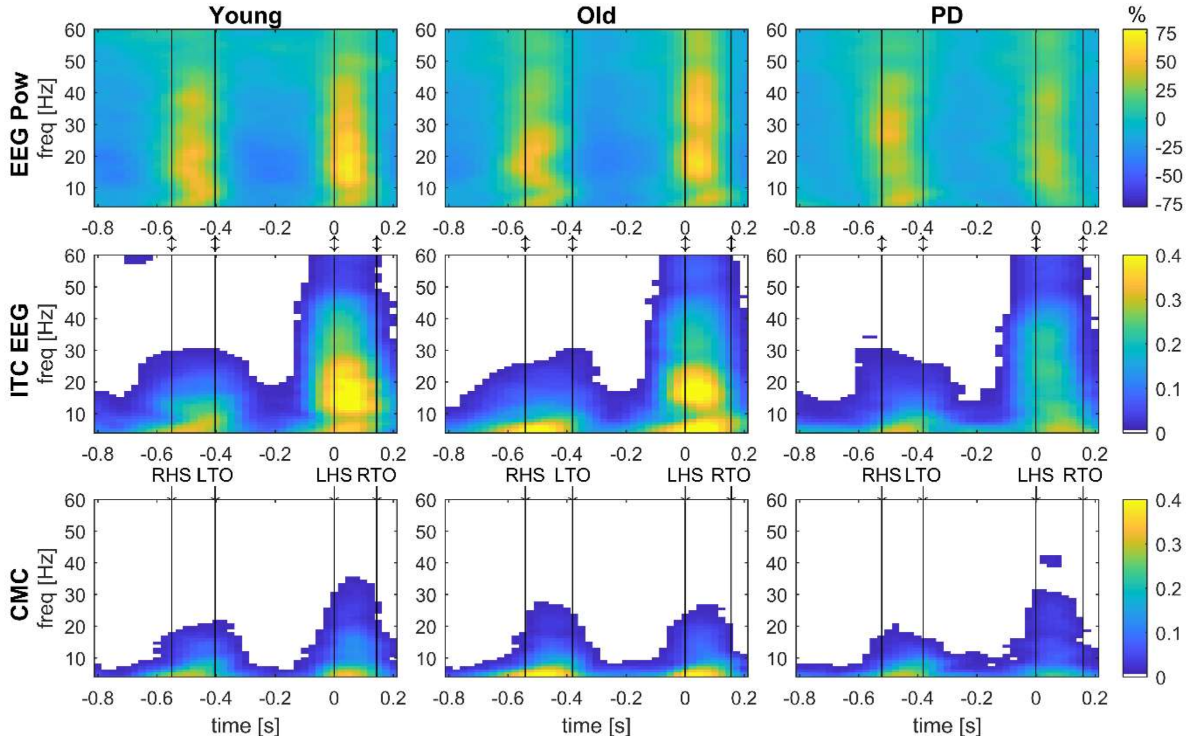

Figure S18. Grand-average time-frequency spectra of artefact power, inter-trial coherence and corticomuscular coherence. Event-related artefact power (EEG Pow, top row), inter-trial coherence of artefacts (ITC EEG, middle row) and corticomuscular coherence (CMC, bottom row) acquired from bipolar artefact-EEG signals of the right sensorimotor cortex (C4-F4) and EMG from the left TA during overground walking in the young, old and PD groups. EEG power shows the percent change from the average. Coherence values (ITC and CMC) are thresholded: average coherence values below the 95% CI are set to zero (white). The x-axis shows the time in seconds relative to heel strike (t=0) of the left foot and the y-axis the frequencies in Hz. Black vertical lines indicate the footswitch events. LHS, left heel strike; LTO, left toe-off; RHS, right heel strike; RTO, right toe-off.

The results of the time-frequency analyses on the ICs and on the artefact components (figures S15 to S18) show that, compared to the processed EEG (e.g. Figure 1 in the main article), the artefact data have higher power (≥75%) and inter-trial coherence (≥0.4). It is also more

broadband at beta frequencies during double support compared to the EEG data shown in Figure 1 (≤50% for power, ≤0.35 for inter-trial coherence). Thus, while the data processing has been effective in removing and reducing the influence of artefacts, the EEG data presented in the main article cannot be assumed to be completely artefact-free and that a cautious interpretation of the results is warranted.

References

Chang C-Y, Hsu S-H, Pion-Tonachini L, and Jung T-P. Evaluation of Artifact Subspace Reconstruction for Automatic Artifact Components Removal in Multi-channel EEG Recordings. *IEEE Transactions on Biomedical Engineering* 1-1, 2019.

Delorme A, Palmer J, Onton J, Oostenveld R, and Makeig S. Independent EEG Sources Are Dipolar. *PLoS ONE* 7: e30135, 2012.

Hsu S-H, Pion-Tonachini L, Palmer J, Miyakoshi M, Makeig S, and Jung T-P. Modeling brain dynamic state changes with adaptive mixture independent component analysis. *NeuroImage* 183: 47-61, 2018.

Wagner J, Martinez-Cancino R, Delorme A, Makeig S, Solis-Escalante T, Neuper C, and Mueller-Putz G. High-density EEG mobile brain/body imaging data recorded during a challenging auditory gait pacing task. *Scientific data* 6: 211, 2019.
